# Effects of osteochondral defect size on cartilage regeneration using a double-network hydrogel

**DOI:** 10.1186/s12891-017-1578-1

**Published:** 2017-05-22

**Authors:** Kotaro Higa, Nobuto Kitamura, Keiko Goto, Takayuki Kurokawa, Jian Ping Gong, Fuminori Kanaya, Kazunori Yasuda

**Affiliations:** 1 Department of Sports Medicine, Graduate School of Medicine, Hokkaido University, Kita-15, Nishi-7, Kita-ku, Sapporo, 060-8638 Japan; 2 Department of Orthopedic Surgery, Graduate School of Medicine, University of the Ryukyus, Okinawa, Japan; 3 Laboratory of Soft and Wet Matter, Department of Advanced Transdisciplinary Sciences, Faculty of Advanced Life Science, Hokkaido University, Sapporo, Japan; 4 Global Station for Soft Matter, Global Institution for Collaborative Research and Education, Hokkaido University, Sapporo, Japan

**Keywords:** Cartilage repair, Double-network hydrogel, Osteochondral defect, Rabbit model

## Abstract

**Background:**

There has been increased interest in one-step cell-free procedures to avoid the problems related to cell manipulation and its inherent disadvantages. We have studied the chondrogenic induction ability of a PAMPS/PDMAAm double-network (DN) gel and found it to induce chondrogenesis in animal osteochondral defect models. The purpose of this study was to investigate whether the healing process and the degree of cartilage regeneration induced by the cell-free method using DN gel are influenced by the size of osteochondral defects.

**Methods:**

A total of 63 mature female Japanese white rabbits were used in this study, randomly divided into 3 groups of 21 rabbits each. A 2.5-mm diameter osteochondral defect was created in the femoral trochlea of the patellofemoral joint of bilateral knees in Group I, a 4.3-mm osteochondral defect in Group II, and a 5.8-mm osteochondral defect in Group III. In the right knee of each animal, a DN gel plug was implanted so that a vacant space of 2-mm depth was left above the plug. In the left knee, we did not conduct any treatment to obtain control data. Animals were sacrificed at 2, 4, and 12 weeks after surgery, and gross and histological evaluations were made.

**Results:**

The present study demonstrated that all sizes of the DN gel implanted defects as well as the 2.5mm untreated defects showed cartilage regeneration at 4 and 12 weeks. The 4.3-mm and 5.8-mm untreated defects did not show cartilage regeneration during the 12-week period. The quantitative score reported by O’Driscoll et al. was significantly higher in the 4.3-mm and 5.8-mm DN gel-implanted defects than the untreated defects at 4 and 12 weeks (*p* < 0.05). The 2.5-mm and 4.3-mm DN gel implanted defects maintained relatively high macroscopic and histological scores for the 12-week implantation period, while the histological score of the 5.8-mm DN gel implanted defect had decreased somewhat but statistically significantly at 12 weeks (*p* = 0.0057).

**Conclusions:**

The DN gel induced cartilage regeneration in defects between 2.5 and 5.8 mm, offering a promising device to establish a cell-free cartilage regeneration therapy and applicable to various sizes of osteochondral defects.

## Background

Articular cartilage injuries often result in pain and compromised joint functioning, and are considered a risk factor for the development and progression of osteoarthritis [1–3]. This has drawn attention to a growing need to repair damaged cartilage because hyaline cartilage tissue cannot regenerate spontaneously in vivo [4, 5]. To create new cartilage to fill in cartilage defects, reparative procedures including bone marrow stimulation techniques, osteochondral graft transplantation, and autologous chondrocyte implantation have been developed, and have been shown to be clinically effective [4–7]. Cell-based therapies such as autologous chondrocyte implantation hold the promise for cartilage repair to be able to regenerate and restore normal functioning. Currently, an innovative approach using mesenchymal stems cells (MSCs) has been investigated as an alternative among cell based therapies to minimize the problems associated with harvesting normal cartilage tissue because MSCs have the capacity to differentiate into many cell and tissue types given appropriate growth conditions [8–12]. However, there are problems and concerns with these current strategies including donor site morbidity, the need to undergo surgery twice, a long non-weight bearing period, the potential risk of zoonotic transmission, and considerable cost [7, 13–15]. An optimal technique would be one that is less invasive, requires surgery only once, is cost effective, and offers the potential of being highly successful without causing complications. To develop this, there has been increased interest in one-step cartilage repair procedures to treat focal cartilage defects.

In the search for a useful material to develop a cell-free method for cartilage repair, we have studied the chondrogenic induction ability of a PAMPS/PDMAAm double-network (DN) gel composed of poly-(2-Acrylamido-2-methylpropanesulfonic acid) (PAMPS) and poly-(N,N’-dimethyl acrylamide) (PDMAAm) and found it to be a promising candidate. Our previous studies have demonstrated that this DN gel has the potential to induce chondrogenesis in vitro, and that cartilage regeneration is induced in vivo in animal osteochondral defect models [16–22]. These studies have suggested a new strategy to repair osteochondral defects by using the artificially synthesized hydrogel without any cultured cells or mammalian-derived scaffolds. We have demonstrated that this therapeutic strategy is effective not only in patellofemoral joint but also in femorotibial joints in rabbits [22]. In addition, we have established that DN gel with a thickness of only 1.0 mm can induce a hyaline cartilage regeneration similar to that of a 5.0 mm thick DN gel plug [19], which will make less invasive surgery possible. However, using this method, the repair of larger defects has not been systematically studied yet. It is not clear whether the DN gel can be used irrespective of the size of osteochondral defects. If it is established that DN gel plugs are an effective biomaterial in cartilage regeneration irrespective of the defect size, it would be an important finding of clinical relevance because most treatment algorithms are primarily driven by defect size [5, 23]. The purpose of this study was to investigate whether the healing process and the degree of cartilage regeneration induced by the cell-free method using DN gel are influenced by the size of osteochondral defects.

## Methods

### Materials

The PAMPS/PDMAAm DN gel was synthesized using the previously reported two-step sequential polymerization method [24]. 2-acrylamido-2-methyl-1-propanesulfonic acid (AMPS) (Toagosei Co. Ltd., Japan) and N,N’-dimethyl acrylamide (DMAAm) (KOHJIN Co., Ltd., Tokyo, Japan) were used as purchased. Briefly, PAMPS hydrogel was obtained by radical polymerization using N,N’-methylenebisacrylamide (MBAA) (Tokyo Chemical Industry Co., Ltd., Tokyo, Japan) as a cross-linker and 2-oxoglutaric acid (Wako Pure Chemical Industries, Ltd, Osaka, Japan) as an initiator. The monomer concentration was 1 mol/l for PAMPS, 4 mol% for the cross-linker, and 0.1 mol% for the initiator. Aqueous solution containing a monomer, cross-linker, and the initiator was injected into a cell consisting of a pair of glass plates separated by silicone rubber. The cell was irradiated with ultraviolet (UV) light (wave length 365 nm) for about 8 h under argon gas. The DN gel was synthesized by the sequential network formation technique (two-step method). The PAMPS hydrogel (1^st^ network) was immersed in an aqueous solution of 2 mol/L DMAAm, containing 0.1 mol% MBAA, and 0.1 mol% 2-oxoglutaric acid for one day until reaching the equilibrium. The 2^nd^ network (PDMAAm) was subsequently polymerized in the presence of the PAMPS hydrogel by irradiating UV for 8 h between two glass plates under argon gas. After polymerization, the PAMPS/PDMAAm DN gel was immersed in 0.9% NaCl solution for 1 week with the water changed twice daily to remove any un-reacted materials From a 5.0 mm thick PAMPS/PDMAAm DN gel block, we punched out 2.7 mm, 4.5 mm, and 6.0 mm diameter plugs from the gel block.

### Study design

Animal experiments were carried out in the Institute of Animal Experimentation, Hokkaido University School of Medicine under the Rules and Regulation of the Institutional Animal Care and Use Committee of National University Corporation Hokkaido University.

**Fig. 1:**
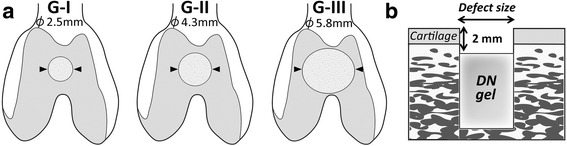
How to induce cartilage regeneration. **a** Cylindrical osteochondral defects of the femoral trochlea of 2.5 mm diameter in Group-I (G-I), 4.3 mm in Group-II (G-II), and 5.8 mm in Group-III (G-III) were created. **b** Schematic cross-section of an osteochondral defect with an implanted plug. Note that a 2-mm deep vacant space from the cartilage surface remained after the surgery

A total of 63 mature female Japanese white rabbits, 6 months old and weighing 3.5 ± 0.6 kg, were used in this study. Animals were randomly divided into 3 groups of 21 animals, each for one defect size. A 2.5-mm diameter osteochondral defect was created in the femoral trochlea of the patellofemoral joint of bilateral knees in Group I (G-I), a 4.3-mm diameter osteochondral defect in Group II (G-II), and a 5.8-mm diameter osteochondral defect in Group III (G-III) (Fig. 1). In the right knee of each animal, a cylindrical DN gel plug was implanted at the bottom of each defect so that a 2-mm deep defect remained above the DN gel after surgery. The depth of 2 mm was chosen because this depth was found to be the most effective to induce the cartilage regeneration in our previous study with rabbits [17, 21]. In the left knee, a defect was left vacant without any implantation to obtain control data. Animals were sacrificed by a lethal dose injection of pentobarbital at 2, 4, and 12 weeks after surgery, and the bilateral knees were harvested. At each period, histological and immunohistochemical evaluations after gross observations were performed. The gross appearance of the regenerated tissue in the defect was evaluated with International Cartilage Repair Society (ICRS) macroscopic evaluation system [7] and the histology was quantitatively evaluated with the O’Driscoll score [25].

### Surgical procedure

The surgery was performed under intravenous anesthesia (pentobarbital, 25 mg/kg). An animal was positioned in dorsal recumbency in a cradle, leaving the hindlimbs free. The bilateral hindlimbs were shaved and prepared in the standard sterile fashion. A midline 3 cm incision was made over the knee joint and subsequently the articular surface of the trochlea was exposed through a medial parapatellar capsulotomy. In the bilateral knees of each animal, an osteochondral defect with a diameter of 2.5 mm (G-I), 4.3 mm (G-II), or 5.8 mm (G-III) was created at the center of the femoral trochlea with a motorized drill, in which the depth of the defect was 7.0 mm. In the right knee of each group, a prepared DN gel plug was press-fitted into the osteochondral defect so that a final vacant space of 2-mm depth from the articular cartilage surface and above the DN gel remained after the surgery. In the left knee, we did not apply any treatment to obtain an untreated control. The incision was closed routinely in layers beginning with the deep fascia. Postoperatively, the animals were returned to their cages (310 × 550 × 320 mm) and allowed to put full weight on their limbs without restrictions on the motion.

### Evaluation methods

#### Gross observation for regenerated tissues

Immediately after the sacrifice, the tissue regenerated in the osteochondral defect was quantitatively evaluated with the ICRS macroscopic evaluation system [7]. The gross appearance of each defect on the femoral condyle was evaluated for *degree of defect repair* (in level with surrounding cartilage, 4 points; 75% repair of defect depth, 3 points; 50% repair of defect depth, 2 points; 25% repair of defect depth, 1 point; and no repair of defect depth, 0 points), *integration to border zone* (complete integration with surrounding cartilage, 4 points; demarcating border < 1 mm, 3 points; three-quarters of graft integrated, one-quarter with a notable border > 1mm width, 2 points; one-half of graft integrated with surrounding cartilage, one-half with a notable border > 1 mm, 1 point; and no contact to one-quarter of graft integrated with surrounding cartilage, 0 points), and *macroscopic appearance* (intact smooth surface, 4 points; fibrillated surface, 3 points; small, scattered fissures or cracks, 2 points; several small or few large fissures, 1 point; and total degeneration of grafted area, 0 points). The maximum total score was 12 points.

### Histological and immunohistochemical examinations

A distal portion of the femur was resected and fixed in a 10% neutral buffered formalin solution for 3 days, decalcified with 50 mM EDTA for 3 weeks, and then cast in a paraffin block. The femur was sectioned parallel to the longitudinal axis, and stained with hematoxylin-eosin (HE) and Safranin-O. For immunohistochemical evaluations, monoclonal antibody (anti-hCL (II), purified IgG, Fuji Chemical Industries Ltd, Toyama, Japan) was used as the primary antibody. Immunostaining was carried out according to the manufacturer instructions using the Envision immunostaining system (DAKO Japan, Kyoto, Japan). Finally, the sections were counterstained with hematoxylin.

The histology of the tissue regenerated in the defect was quantitatively evaluated with the scoring systems reported by the O’Driscoll [25]. Histology was evaluated for *cellular morphology* (hyaline articular cartilage, 4 points; incompletely differentiated mesenchyme, 2 points; and fibrous tissue or bone, 0 points), *Safranin O staining of the matrix* (normal or nearly normal, 3 points; moderate, 2 points; slight, 1 point; and none, 0 points), *surface regularity* (smooth and intact, 3 points; superficial horizontal lamination, 2 points; fissures of 25–100% of thickness, 1 point; and severe disruption including fibrillation, 0 points), *structural integrity* (normal, 2 points; slight disruption including cysts, 1 point; and severe disintegration, 0 points), *thickness* (100% of normal adjacent cartilage, 2 points; 50–100% of normal cartilage, 1 point; and 0–50% of normal cartilage, 0 points), *bonding to the adjacent cartilage* (bonded at both ends of the graft, 2 points; bonded at one end or partially at both ends, 1 point; and not bonded, 0 points), *hypocellularity* (normal cellularity, 3 points; slight hypocellularity, 2 points; moderate hypocellularity, 1 point; and severe hypocellularity, 0 points), *chondrocyte clustering* (no clusters, 2 points; <25% of the cells, 1 point; and 25–100% of the cells, 0 points), and *adjacent cartilage degradation* (normal, no clusters, and normal staining, 3 points; normal cell, mild clusters, and moderate staining, 2 points; mild/moderate hypocellularity and slight staining, 1 point; severe hypocellularity, 0 points). The maximum total score was 24 points.

### Statistical analysis

All data were described as mean and standard deviation values. The mean value of each parameter was compared among the groups using the multi-factor analysis of variance (ANOVA) with the Tukey-Kramer test for post-hoc multiple comparisons. The significance limits were set at *p* = 0.05.

## Results

### Gross observation of the joint surface repair

**Fig. 2:**
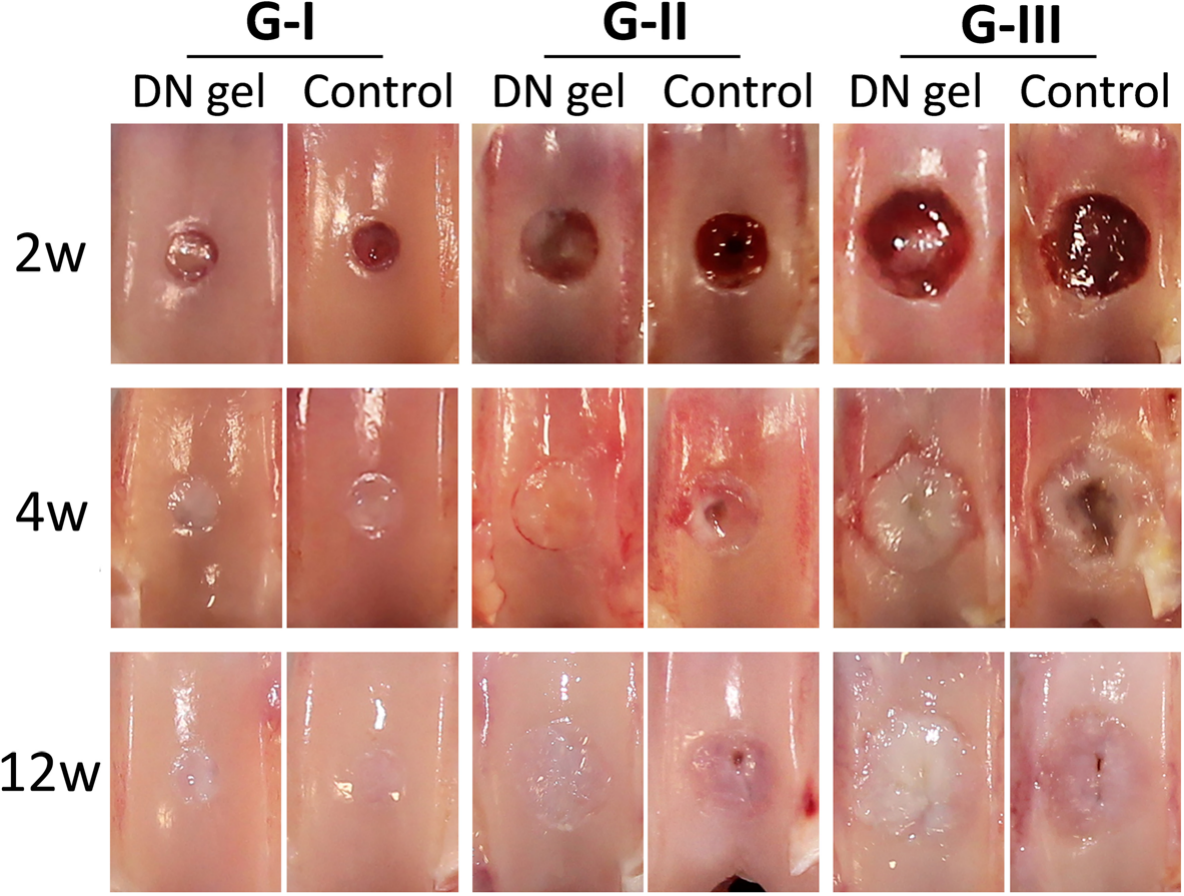
Gross observations of the joint surface at 2, 4, and 12 weeks. At 2 weeks, the defects in G-I and II with the DN gel were insufficiently filled with *pinkish white* tissue and other defects were not filled with regenerated tissue. At 4weeks, the defects in G-I with DN gel and the untreated controls, and G-II and III with DN gel were filled with a white opaque tissue, while the defects in G-II and III of the untreated controls were partly filled with *white* or *reddish*, patchy tissue with an irregular surface. At 12 weeks, the defects in G-I with DN gel and the untreated controls, and G-II and III with DN gel were filled with a white opaque tissue, while the surface of the regenerated tissue in G-III with DN gel were somewhat irregular. The defects in the G-II and III untreated controls were insufficiently filled with *white* or *reddish*, patches of tissue with an irregular surface

In the gross observations, the knee joint did not show any severe inflammation or apparent fibrosis in any of the animals. At 2 weeks, the 2.5-mm untreated defect (G-I) was filled with somewhat white tissue, while the 2.5-mm DN gel-implanted was filled with a pinkish tissue. Further, the 4.3-mm and 5.8-mm defects (G-II and III) were insufficiently filled with a somewhat reddish tissue containing patches of white tissue with or without the DN gel implantation. The surface of these defects appeared irregular and was partly depressed in the shape (Fig. 2).

At 4 weeks, both the 2.5-mm DN gel-implanted and untreated defects (G-I) were completely filled with a regenerated white tissue, with the border of the defects still distinct. The 4.3-mm DN gel-implanted defects (G-II) were filled with a somewhat white tissue, while the 4.3-mm untreated defects (G-II) were filled, but not fully, and displayed an irregular surface. The 5.8-mm DN gel-implanted defects (G-III) were filled with a somewhat white tissue with the border of the defects clearly distinct. All of the 5.8-mm untreated defects (G-III) were filled with tissue which had an uneven surface and a crater in the central region (Fig. 2).

At 12 weeks, both the 2.5-mm DN gel-implanted and untreated defects (G-I) were completely filled with a regenerated white tissue without a clear border. The defects had a smooth surface and coloration closer to that of surrounding normal cartilage. The 4.3-mm DN gel-implanted defects (G-II) were also filled with a white tissue with a smooth surface, while the 4.3-mm untreated defects (G-II) were insufficiently filled with a white tissue, pinkish in places. The 5.8-mm DN gel-implanted defects (G-III) were filled with a white tissue with the border of the defect still distinct. The 5.8-mm untreated defects (G-III) were filled with a white tissue, pinkish in places, and with a depression in the defects (Fig. 2).

### Histological and immunohistochemical evaluations

**Fig. 3:**
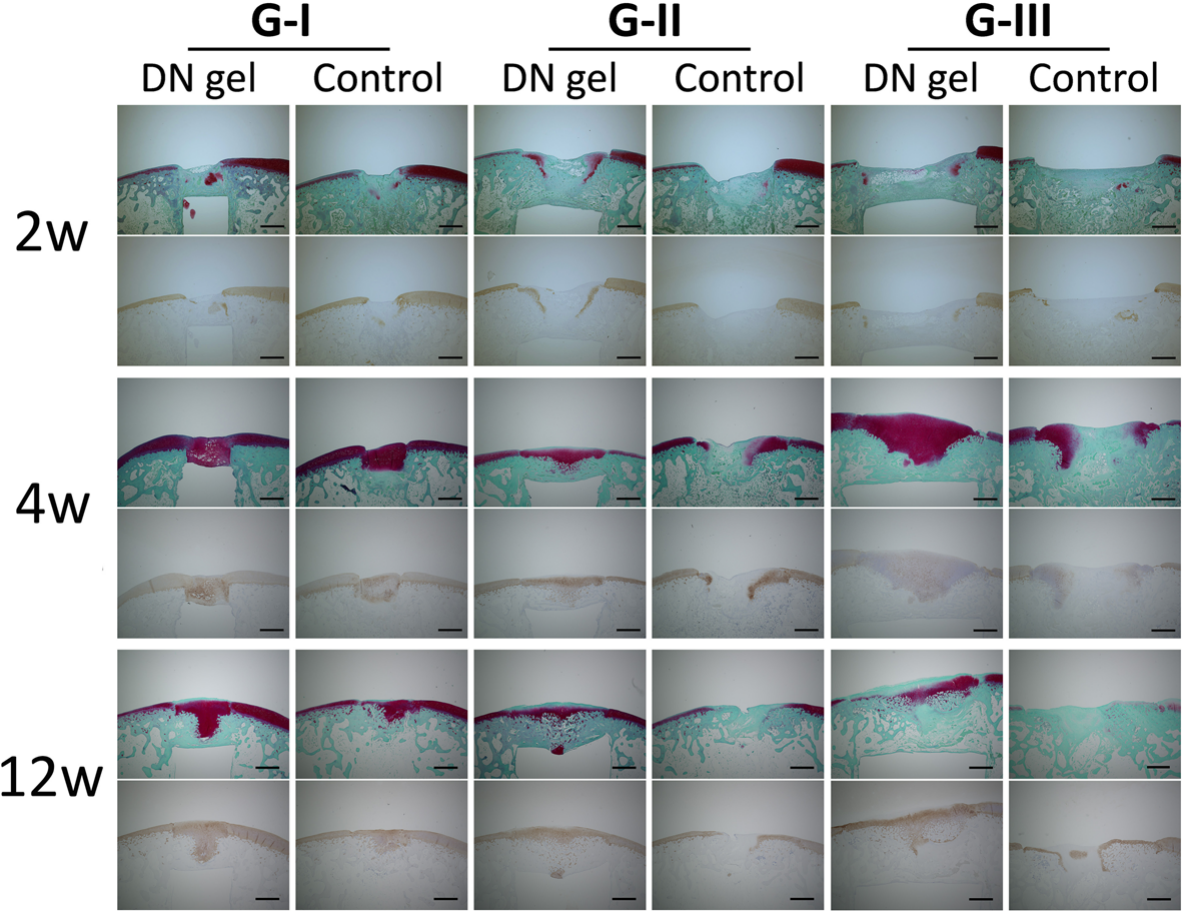
Safranin-O staining and type-2 collagen immunohistochemical staining at 2, 4, and 12 weeks. At 2 weeks, all defects were filled with fibrous tissue. In the defects in G-I with DN gel and the untreated controls, and G-II with DN gel, some tissue was stained with Safranin-O. At 4 weeks, the defects in G-I with DN gel and the untreated controls and G-II and III with DN gel were filled with a sufficient volume of proteoglycan-rich tissue with regenerated sub chondral bone tissue. At 12 weeks, the histology showed similar findings to that at 4 weeks in all groups, while the area and degree of the Safranin-O staining were reduced in the defects in G-III with DN gel. Immunohistochemical staining for type-2 collagen were consistent with the Safranin-O staining. *Blac*k scale bar = 1 mm

At 2 weeks, all defects were primarily filled with fibrous or fibrous cartilaginous tissue regardless of the defect size or the implantation of the DN gel. Small areas at the peripheral regions were found to be partly stained with Safranin-O (Fig. 3).

**Fig. 4:**
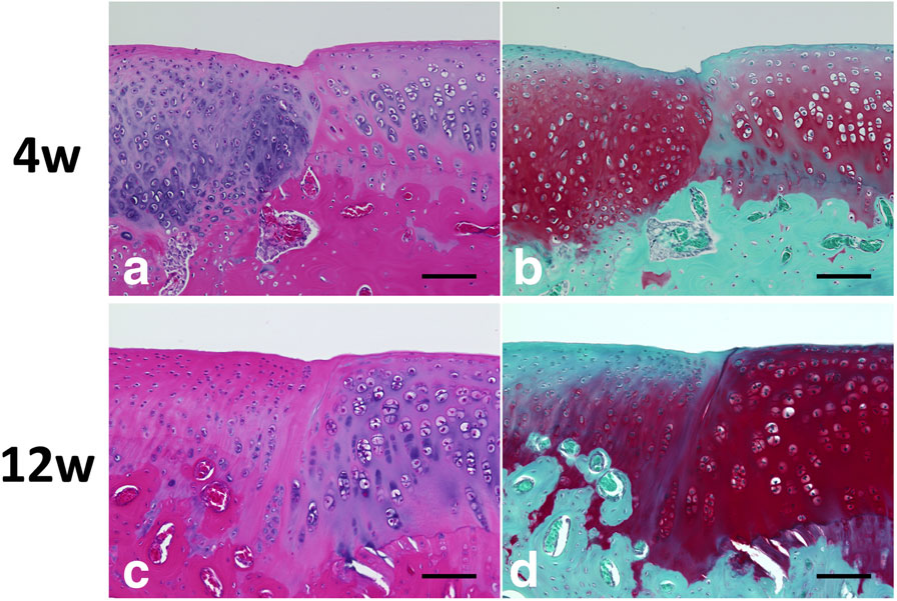
High-magnification (20× original magnification) histological observations at the lateral border area of DN gel-implanted defects of G-II at 4 and 12 weeks (**a**, **c**: HE, **b**, **d**: Safranin-O). The regenerated cartilage was integrated with the adjacent cartilage. *Black* scale bar = 100 μm

At 4 weeks, both the 2.5-mm DN gel-implanted and untreated defects (G-I) were completely filled with a proteoglycan-rich tissue positive to Safranin-O staining. The 4.3-mm and 5.8-mm DN gel implanted defects (G-II and III) were filled with regenerated tissue positive to Safranin-O staining and with regenerated subchondral bone tissue, while the 4.3-mm and 5.8-mm untreated defects (G-II and III) were primarily filled with fibrous and fibrous cartilaginous tissue including tissue partly stained with Safranin-O at the peripheral region of the defect (Fig. 3). High magnification histology showed that round cells rich in cytoplasm were scattered in a proteoglycan-rich matrix in the regenerated tissue of the DN gel-implanted defect and the regenerated cartilage was well integrated with the adjacent cartilage without any significant gaps at 12 weeks (Fig. 4).

**Fig. 5:**
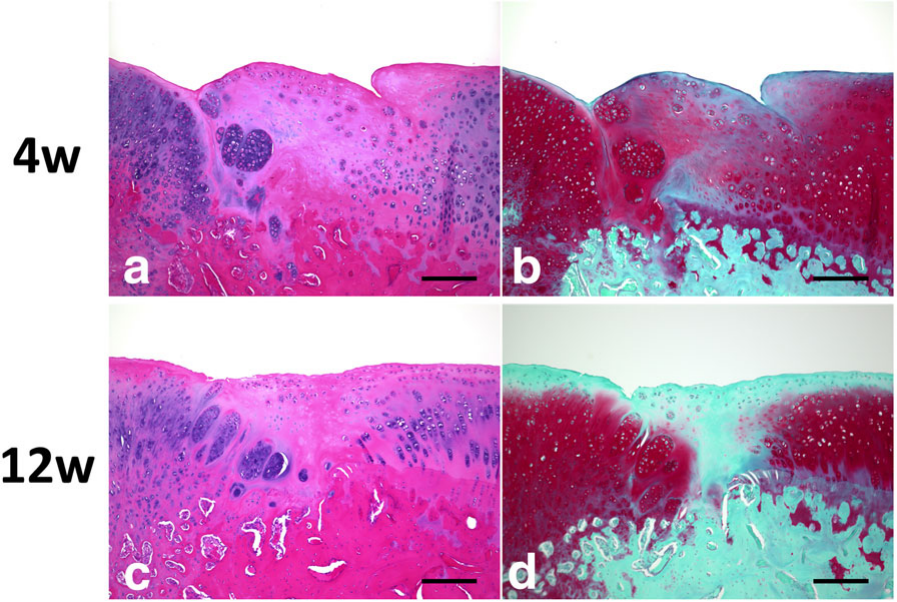
High-magnification (10× original magnification) histological observations at the lateral border area of DN gel-implanted defects of G-III at 4 and 12 weeks (**a**, **c**: HE, **b**, **d**: Safranin-O). *Black* scale bar = 200 μm

At 12 weeks, the histology observations of all groups were similar to that at 4 weeks, with the area and degree of the Safranin-O staining reduced. Specifically, the colouring in the Safranin-O staining was weaker in the 5.8-mm DN gel implanted defects (G-III) compared with that at 4 weeks (Fig. 3). The border area between the regenerated cartilage and adjacent cartilage was stained with Safranin-O but the surface was irregular at 4 weeks, which became less stained with Safranin-O at 12 weeks (Fig. 5). No Safranin-O staining was observed in the 4.3-mm and 5.8-mm untreated defects (G-II and III) (Fig. 3).

Immunohistochemical staining for type-2 collagen were consistent with the Safranin-O staining (Fig. 3). The void space inside the bone indicated the presence of the DN gel, which was removed at the preparation of the histological section.

### Quantitative evaluations of the gross appearance and histology using the grading scales

**Fig. 6:**
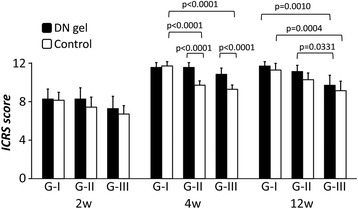
The ICRS (International Cartilage Repair Society) score for quantitative macroscopic evaluations. There were no statistically significant differences in the scores of G-I, II, and III at 2 weeks. At 4 weeks, the scores of G-II and III with DN gel were significantly higher than the untreated controls in all groups (*p* < 0.0001). The scores in the G-I untreated controls were significantly higher than those in the G-II and III untreated controls (*p* < 0.0001). At 12 weeks, there were no significant differences between the DN gel and untreated controls in any group. There was a significant difference in the score between the G-I with DN gel and G-III with DN gel, and between G-II with DN gel and G-III with DN gel (*p* = 0.0010, *p* = 0.0331, respectively). And the scores in the G-I untreated controls were significantly higher than those in the G-III untreated controls (*p* = 0.0004)

For the ICRS score, the ANOVA demonstrated that there was a statistically significant difference in the effect of the DN gel (*p* < 0.0001), the effect of the defect size (*p* < 0.0001), and the effect of the duration after the surgery (*p* < 0.0001). At 2 weeks, there were no significant differences in the score between the DN gel-implanted and untreated defects and among any of the groups with different defect sizes. At 4weeks, the score was significantly higher in the 4.3-mm and 5.8-mm DN gel-implanted defects (G-II and III) than the untreated defects (*p* < 0.0001). The score in 2.5-mm untreated defect (G-I) was significantly higher than that in 5.8-mm defect (G-III) (*p* < 0.0001). At 12 weeks, there were no significant differences between the DN gel-implanted and untreated defects in any of the groups with different defect sizes. There was a significant difference in the score between the 2.5-mm and 5.8-mm DN gel implanted defects (G-I and III) (*p* = 0.0010) and between the 4.3-mm and 5.8-mm DN gel implanted defects (G-II and III) at 12 weeks (*p* = 0.0331). The score of 2.5-mm untreated defects (G-I) was significantly higher than that in the 5.8-mm defects (G-III) (*p* = 0.0004) (Fig. 6).

**Fig. 7:**
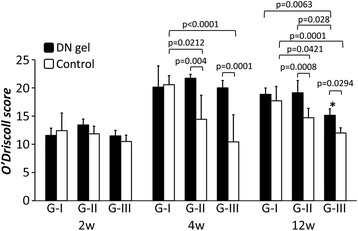
The O’Discoll score for quantitative histological evaluation. At 2 weeks, there were no statistically significant differences in the score in any of the three groups. At 4weeks, the scores in G-II and III with DN gel were significantly higher than in the untreated controls (*p* = 0.0040, *p* = 0.0001, respectively). The scores in the G-I untreated controls were significantly higher than those in the G-II and III untreated controls (*p* = 0.0212, *p* < 0.0001, respectively). At 12 weeks, the scores in G-II and III with DN gel were significantly higher than in the untreated controls (*p* = 0.0008, *p* = 0.0294, respectively). The scores in G-I and II with DN gel were significantly higher than those in G-III with DN gel (*p* = 0.0063, *p* = 0.0280, respectively). The scores in the G-I untreated controls were significantly higher than those in the G-II and III untreated controls (*p* = 0.0421, *p* = 0.0001, respectively). *The scores in G-III with DN gel at 12 weeks were significantly lower than those in G-III with DN gel at 4 weeks. (*p* = 0.0057)

For the O’Driscoll score, the ANOVA demonstrated that there was a statistically significant difference in the effect of the DN gel (*p* < 0.0001), the effect of the defect size (*p* < 0.0001), and the effect of the duration after the surgery (*p* < 0.0001). At 2 weeks, there were no significant differences in the scores of the DN gel-implanted and untreated defects or among the defect size groups. At 4weeks, the O’Driscoll score was significantly higher in the 4.3-mm and 5.8-mm DN gel-implanted defects (G-II and III) than the untreated defects (*p* = 0.0040, *p* = 0.0001, respectively). And the scores in 2.5-mm untreated defects (G-I)) were significantly higher than those in the 4.3-mm and 5.8-mm untreated defects (G-II and III) (*p* = 0.0212, *p* < 0.0001, respectively). At 12 weeks, the score was significantly higher in the 4.3-mm and 5.8-mm DN gel-implanted defects (G-II and III) than the untreated defects (*p* = 0.0008, *p* = 0.0294, respectively). There was a significant difference in the score between the 2.5-mm and 5.8-mm DN gel implanted defects (G-I and III) (*p* = 0.0063) and between the 4.3-mm and 5.8-mm DN gel implanted defects (G-II and III) at 12 weeks (*p* = 0.0028). And the scores in 2.5-mm untreated defects (G-I) were significantly higher than those in 5.8-mm defect (G-III) (*p* < 0.0001). Both the 2.5-mm and 4.3-mm DN gel implanted defects (G-I and II) maintained relatively high scores during the 12-week implantation period, while the O’Driscoll score of the 5.8-mm DN gel implanted defect (G-III) was significantly lower at 12 weeks than at 4 weeks (*p* = 0.0057) (Fig. 7).

## Discussion

The present study demonstrated that DN gel induced cartilage regeneration in defects which were intentionally created with a 2-mm deep vacant space above implanted DN gel was feasible in defect sizes between 2.5 and 5.8 mm diameter in the rabbit model here. The 2.5-mm and 4.3-mm DN gel implanted defects maintained relatively high macroscopic and histological scores for the 12-week implantation period of observation, while the O’Driscoll score of the 5.8-mm DN gel implanted defect decreased slightly but statistically significantly at 12weeks. Based on the findings, it may be concluded that the DN gel is an excellent cartilage regeneration inducing material independent of defect size. However, it is possible that the characteristics of the regenerated tissue and cells in the defect treated with DN gel may be different in the defect groups because the smaller defects maintained a relatively good quality of cartilage tissue but the larger defects did not.

Defect size is a very important factor in both the clinical application and the biological aspect. A number of surgical techniques for treatment of cartilage injuries have been reported such as bone marrow stimulation, osteochondral graft transplantation, and autologous chondrocyte implantation. The surgical indication of these procedures usually relies on the defect size of cartilage injury. The defect size of 2 cm^2^ is used as a clinical threshold parameter in determining which surgical procedure to adopt for a lesion [5, 23]: Bone marrow stimulation techniques such as microfracture is usually applied to defect sizes smaller than 2 cm^2^, while autologous chondrocyte implantation is used for larger defects. This strategy is reasonable because cartilage injuries have different healing responses depending upon the defect size. Based on animal studies using a rabbit cartilage defect model, defect sizes of 3 mm or smaller has a high potential for spontaneous healing with hyaline-like cartilage tissue in the early stage of the reparative process [26, 27]. We consider that the findings in the present study are relevant to these previous studies, that the smaller untreated defect healed spontaneously but the larger untreated defects did not, as shown in the results that cartilage regeneration was spontaneous in the 2.5-mm defect.

Based on the previous studies reporting cartilage regeneration using a DN gel, the depth of the defect remained after the DN gel implantation is a critical factor on the cartilage regeneration induced by the DN gel: An approximately 2-mm deep vacant space above the DN gel is the most effective for cartilage regeneration in a mature rabbit femoral trochlea osteochondral defect model [17, 19–21], this suggests that the appropriate size of space is very important for regeneration of high quality cartilage tissue. The present study used a 2-mm depth model and showed cartilage regeneration in the vacant space independent on the defect size at 4 weeks. However, the results at 12 weeks varied somewhat among the defect size groups. We speculate that the biomechanical condition influenced the regeneration process of each defect size group because the biomechanical condition is important on chondrogenic differentiation of MSCs [28–30]. Because the concept of this method using the DN gel is based on recruiting cells onto the DN gel surface in the vacant space, there may be an appropriate depth dependent upon the defect size. For example, the 5.8-mm DN gel implanted defect may demonstrate a better result and may maintain a good quality of cartilage tissue at 12 weeks if the depth of the defect were shallower. Further studies are needed to clarify the interaction between the size and depth of the defect with regard to the quality of cartilage regeneration induced by the DN gel.

There are some limitations to this study. First, we used a rabbit model in this study, and there is a need to conduct experimental studies with a large animal model in order to fully evaluate the pre-clinical efficacy. The second limitation is that we used a patellofemoral joint model in this study. This model has commonly been used to evaluate new procedures in cartilage regeneration experiments, but the results obtained cannot be simply applied to the clinical field because different results might be obtained in the femorotibial joint model. The third limitation is that we performed only 12-week of observations of the regenerated cartilage. The fourth limitation is that we evaluated the regenerated cartilage tissue only histologically: We did not evaluate the cell viability, gene expression, or extracellular matrix change in the regenerated cartilage in the present study. Despite the relatively good histological results, the genetic characteristics of the regenerated cartilage tissue may be different from the native cartilage tissue. The fifth limitation is that no biomechanical evaluations of the regenerated cartilage were performed.

An innovative strategy with the DN gel implantation for cartilage repair without cell culture is proposed. In addition, this strategy is unique in that the DN gel generated hyaline-like cartilage without fully filling the defect. We speculate that an appropriate physical environment for cartilage regeneration was produced in the vacant space by implanting the DN gel at the bottom of the defect. This DN gel is a very mechanically stable material in vivo: The DN gel shows a fracture strength of 3.1 MPa, whereas the fracture strength of the single network gels ranges from a few to several hundreds of kilopascals [24, 31]. Because the biomechanical microenvironment is known to be important in chondrogenic differentiation of MSCs [8–10, 28–30], the DN gel may have the potential to differentiate the MSCs contained in blood clots into chondrocytes. We can expect this strategy to solve the various problems and concerns in the current strategies [7, 13–15] and change the treatment algorithm by widening the indication for treatment of larger defects. Concerning the safety of the DN gel as a biomaterial, we conducted a pellet implantation test into the para-vertebral muscle for 6 weeks [32], according to the guidelines for biological evaluation of the safety of biomaterials, which has been published by the Ministry of Health, Labour and Welfare, Japan. The DN gel implantation did not induce significant inflammation during the implantation periods [32]. We also cultured ATDC5 cells on the DN gel and no harmful effects due to the DN gel surface were detected [16, 21]. The results of the present study have added important information to realize the benefits of this innovative strategy with the DN gel.

## Conclusions

The present study demonstrated that the DN gel without the use of exogenous cells induced cartilage regeneration in defects from 2.5 to 5.8 mm deep. This therapeutic strategy is new and a completely different concept from the current progressive strategies and offers the possibility to change the treatment algorithm by widening the indication for treatment of larger defects. The DN gel composed of PAMPS and PDMAAm is a promising device to enable cell-free cartilage regeneration with potential applications to various defect sizes.

## Abbreviations

ANOVA: Analysis of variance
DN: Double-network
EDTA: Ethylene diamine tetraacetic acid
HE: Hematoxylin-eosin
ICRS: International Cartilage Repair Society
MBAA: N,N’-methylenebisacrylamide
MSCs: Mesenchymal stems cells
PAMPS: Poly-(2-Acrylamido-2-methylpropanesulfonic acid)
PDMAAm: Poly-(N,N’-dimethyl acrylamide)
UV: Ultraviolet
